# Effect of time-dependent ibuprofen administration on the post operatory after impacted third molar extraction: a cross-over randomized controlled trial

**DOI:** 10.1007/s10006-022-01104-8

**Published:** 2022-08-03

**Authors:** Fabián Pérez-González, Mohammad Abusamak, Luis Miguel Sáez-Alcaide, Jesus Torres García-Denche, Faleh Ahmad Tamimi Marino

**Affiliations:** 1 Faculty of Dentistry, Department of Dental Clinical Specialties, University Complutense of Madrid, Plaza Ramón y Cajal S/N, 28040 Madrid, Spain; 2 Faculty of Dental Medicine and Oral Health Sciences, McGill University, Montreal, Canada; 3 College of Dental Medicine, Qatar University, Doha, Qatar

**Keywords:** Posology, Chronotherapy, Ibuprofen, Extraction, Third molar

## Abstract

**Purpose:**

To evaluate time-dependent administration of ibuprofen in a lower third molar extraction model.

**Methods:**

Eleven patients requiring bilateral surgical removal of lower third molars were recruited and randomized into a blinded crossover randomized controlled trial. For 3 days after surgery, the control group was prescribed ibuprofen 400 mg every 8 h. On the other hand, the experimental group received also ibuprofen 400 mg at breakfast and lunch, replacing the dinner intake with a placebo. Pain measurements (Visual Analog Scale from 0 to 10) were recorded at baseline, 24, 48, and 72 h postoperatively. Facial swelling and trismus were also measured at baseline, 24, and 72 h postoperatively.

**Results:**

Postoperative swelling and pain perception did not show significative difference between the control and experimental groups at 24, 48, and 72 h. Trismus was significantly lower in the control group than in the experimental group at 72 h postoperatively (*p* = 0.008). Rescue medication consumption seemed to be comparable between groups.

**Conclusion:**

Eliminating night time ibuprofen might be insignificant for pain control after third molar extraction.

## Introduction

Third molar (wisdom tooth) extraction, both prophylactically and due to odontogenic infection, is one of the most common surgical interventions in the world [1–4]. Postoperatively, such surgery is associated with pain, swelling, or/and trismus [1, 3, 5–7]. Third molar extraction also causes high anxiety levels, increases morbidity, and impacts patients’ quality of life [8].

In addition, wisdom teeth surgery is estimated to cost $3 billion per year in the USA alone, which places a burden on healthcare systems, and a proper pain management with a correct drug dosage could result in savings of $130 M per patient and reduce the back to work after health problems from 66 to 27% [8, 9].

Several medications are routinely prescribed for pain management after third molar extraction such as acetaminophen, opioids, and non-steroidal anti-inflammatory drugs (NSAID). Acetaminophen is insufficient for managing moderate to severe postoperative pain, and opioids can be addictive and cause constipation. NSAID, which are commonly prescribed, are indeed efficient in managing postoperative pain, but they have several adverse effects such as gastrointestinal complications and delayed bone healing [10–12]. Ibuprofen is the most used NSAID [13]. It is a 2-propionic acid derivate that was discovered in the 1960s by the British Boots Group, and it is a peripheral-acting analgesic with a high anti-inflammatory effect that reversibly inhibits COX-1 and COX-2 [14]. All organisms have a 24-h cycle pattern known as *circadian rhythm* that controls and modulates a wide range of their biological activities [15]. These rhythms have two main phases: an active or diurnal phase and a rest or nocturnal phase [16, 17]. The circadian rhythms also regulate the bone metabolism in 24-h oscillations affecting its formation, resorption [18–20], and even its healing after bone fracture [19, 21].

Moreover, inflammatory mediators have been described as day-time dependent [15, 22]. For example, pro-inflammatory cytokines such as IL-1 β and IL-6 are higher during the active phase, while the anti-inflammatory cytokines reach their peak during the resting phase [23, 24]. Further studies suggested that cytokines play a major role in pain perception, and thereafter, pro-inflammatory cytokine modulation is vital for pain management and tissue recovery [25].

Different clinical studies suggested that timing of drug administration (i.e., *chronotherapy*) with the circadian rhythm of the body could lead to superior pain management and fewer side effects [26]. For example, acetaminophen action is better during the night, and NSAIDs absorption and its anti-inflammatory effect is higher when it is administered during the active phase of the circadian rhythms [27]. In fact, it has been shown that NSAID intake during the active phase (diurnal) could modulate the synthesis and release of cytokines by the cyclooxygenase inhibition, causing a decrease in the pro-inflammatory cytokines (like IL-1 β) and promoting the anti-inflammatory cytokines (such as IL-13 and IL-14) [28]. Furthermore, COX-1 and COX-2 activity follows a circadian pattern that regulates bone metabolism after bone tissue damage. Therefore, understanding these circadian variations in NSAID pharmacokinetics (absorption and maximal effect), cytokine release and COX activity would imply the possibility of establishing a chronotherapeutic treatment that would maximize the effect of NSAIDs while reducing their postoperative side effect [23, 29, 30]. This was clearly demonstrated in an animal study evaluating NSIAD chronotherapy after bone fracture in mice. They concluded that NSAID administration during the active phase revealed a superior bone healing outcome, while the administration during the rest phase showed a prolonged inflammatory phase, subsequently decreasing postoperative recovery [28].

In the recent years, it has been proposed that the pharmacokinetic of these drugs could be influenced by the administration time. This implies that coordinating the drug intake time with the circadian clock, a.k.a. *chronotherapy*, could help improve treatment effectiveness.

This pilot study aims to evaluate the effect of NSAID *chronotherapy* in postoperative recovery after third molar extraction related to the swelling, trismus, and pain scores, compared to a conventional NSAID administration regimen. The main hypothesis is that NSAID dosage according to the circadian rhythms results in a similar or better postoperative recovery.

## Material and methods

### Study design

This randomized, double-blinded, placebo-controlled, crossover design pilot study was conducted following the CONSORT [31] guidelines and was carried out at the Oral Surgery Department of the Faculty of Dentistry at the Complutense University of Madrid (UCM). The study was evaluated and approved by the Research Ethics Committee at the San Carlos Clinical Hospital of Madrid, Spain (Trial registration code CEIC 19/216-R_M_BNI.) and the *Agencia Española del Medicamento y Productos Sanitarios* (AEMPS: *Spanish Agency of Drugs and Sanitary Products*, EUDRACT number 2019–000,736). Informed consent was obtained from all participants in writing prior to conducting the research, and the principles of the Declaration of Helsinki for research involving human subjects were followed. The trial was registered with the ClinicalTrials.gov number NCT05126264 in October 2021.

### Participants and inclusion criteria

Patients were accepted in the clinical trial from those who attended the Oral Surgery Department at the Dentistry Faculty of the Complutense University of Madrid between September 2019 and December 2020 with needs of lower third molar extraction. The screening examination was performed by a 2nd-year resident program in Oral Surgery and Implant Dentistry (FPG) and included a medical and dental questionnaire and a standardized panoramic radiograph made at the Dental Radiology Service, Faculty of Dentistry, Complutense University of Madrid (CS 9300®, Carestream Dental, Atlanta, GA, USA). Healthy males and females aged between 18 and 35 years old who presented with impacted bilateral lower third molar with similar surgical difficulty [32] were included in our study. Patients who refused to give consent, to undergo surgery, or who were unable to return for evaluation were excluded. In addition, patients with a history of gastrointestinal disease, pregnant or lactating, and with active periodontal disease were excluded.

### Blinding and randomization

To reduce bias for this pilot study, both patients and surgeon were blinded to treatment group allocation. Second-year surgical resident performed all the surgical procedure (FPG). Both third molar side and medication (3 times ibuprofen or 2 times ibuprofen + placebo) randomization were performed by the main investigator (JTGD) using a coin flip. Other than providing medications, the investigator did not have any contact with the study’s participants or involvement in data collection. Data analysis was performed by an independent investigator (MA).

### Surgical procedure

All surgical procedures were conducted by a single surgeon (FPG) at 9:00 am in all the cases. A minimum of 1 month for washing up was left between one surgery and the intervention on the contralateral side. Local anesthesia consisting of 4% articaine with adrenaline 1:100,000 was administered for the inferior alveolar, lingual, and buccal nerves (Ultracaine®, Normon SL, Madrid, Spain). An intrasulcular incision from the lower first molar with a vertical releasing incision in the ramus was made, and then a mucoperiosteal flap was elevated. A tungsten carbide bur with a surgical handpiece was used to perform bone removal, and when necessary, also a Lindemann bur was used to section the third molar. After the tooth extraction, bony edges were smoothened, and socket was washed with copious use of saline solution. Then, the flap was sutured with simple interrupted sutures using 4.0 Supramid (Proclinic®, Zaragoza, Spain). Surgery time, surgery difficulty according to Parant scale [32], and surgical complications were also recorded. Patients were prescribed amoxicillin 750 mg to be taken every 8 h for 7 days postoperatively. The control group received one 400 mg ibuprofen capsule every 8 h for 5 days, while the experimental group received one 400 mg ibuprofen capsule in the morning and evening and one placebo capsule in the night for 5 days.

### Study outcome measures

According to our primary outcome, pain measurements (Visual Analog Scale from 0 to 10) were recorded at baseline, 24, 48, and 72 h postoperatively. As a secondary outcome, facial swelling parameters such as distance from Tragus to Pogonin (Tg-Pg) [33] and trismus (i.e., interincisal distance) were evaluated at baseline, 24, and 72 h postoperatively. The rescue medication (RM) (acetaminophen 650 mg) consumed by patients was also recorded (day/time/number of pills).

### Statistical analysis

Data were entered on a spreadsheet (MS Excel 2007, Microsoft Inc., Redmond, WA, USA) until the end of the trial and analyzed with R statistical program (4.0.2) by an independent investigator (MA). The significance level chosen for all statistical tests was *p* < 0.05. Descriptive statistics were calculated for all variables (frequency, median, and IRQ range). After testing for normality, non-parametric tests were used. For the quantitative variables, Wilcoxon signed rank test was conducted, and as to qualitative variables, McNemar’s chi-squared test was performed.

## Results

In this RCT, 11 patients, median age of 21 years (*IQR* = 20.00, 21.50) were recruited, evaluated, and included in our analysis. Baseline parameters are presented in Table 1. With the exception of extracted tooth, impaction type, and surgery duration, all demographic variables seem to be equally distributed among experimental groups, as this study followed a split-mouth model. From the sample, 10 patients were caucasic, and only one patient was asiatic. Tooth number 48 was predominantly extracted in the control group (10/11 cases). On the other hand, surgeries lasted longer in the treatment group. Almost all surgeries were performed on partially impacted teeth (10/11 cases). Medical history and medication were not determined for the study.

**Table 1:** Demographics and baseline parameters

Variables	Treatment	Control
Position winter (%)		
Distal	2 (18.2)	0 (0.0)
Horizontal	3 (27.3)	2 (18.2)
Mesial	5 (45.5)	4 (36.4)
Vertical	1 (9.1)	5 (45.5)
Surgery description (%)		
I—Flap only	0 (0.0)	1 (9.1)
II—Flap and osteotomy	7 (63.6)	7 (63.6)
III—Flap, osteotomy, and coronal sectioning	3 (27.3)	1 (9.1)
IV—Flap, osteotomy, and coronal and radicular sectioning	1 (9.1)	2 (18.2)
Swelling baseline (median [IQR])—mm	151.00 [146.50, 160.00]	152.00 [150.50, 160.00]
Trismus baseline (median [IQR])—mm	45.00 [40.00, 55.50]	45.00 [41.00, 53.50]
VAS pain baseline (median [IQR])	0.00 [0.00, 0.00]	0.00 [0.00, 0.00]
Surgery duration (median [IQR])—mins	13.45 [11.57, 15.00]	9.06 [6.36, 11.35]
Quirurgical complications (%)		
Flap tear	0 (0.0)	1 (9.1)
No	11 (100.0)	10 (90.9)
Postoperative complications (%)		
No	10 (90.9)	11 (100.0)
Temporary paresthesia buccal nerve	1 (9.1)	0 (0.0)

**Fig. 1:**
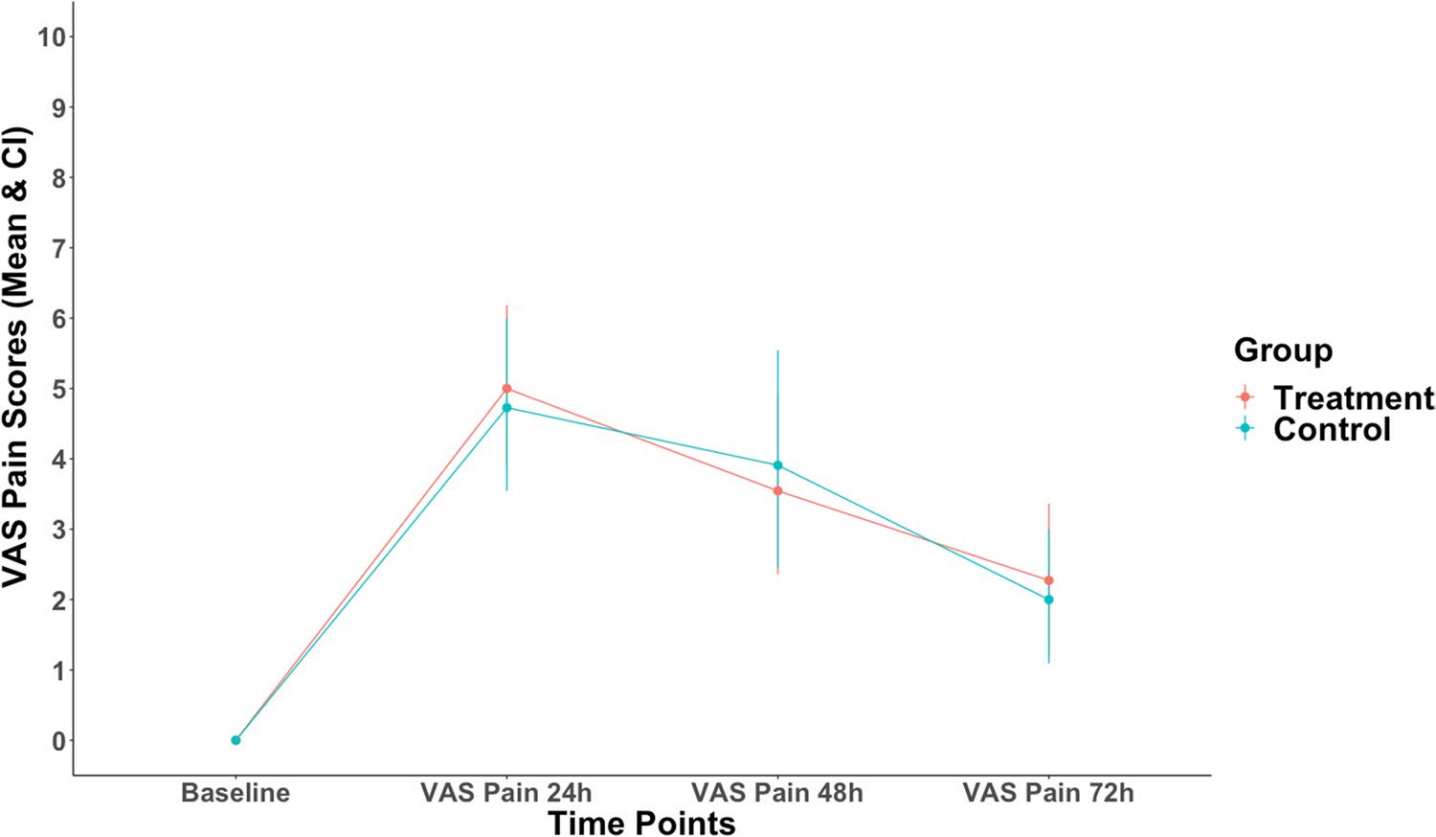
Postoperative VAS pain measures between treatment and control groups

**Fig. 2:**
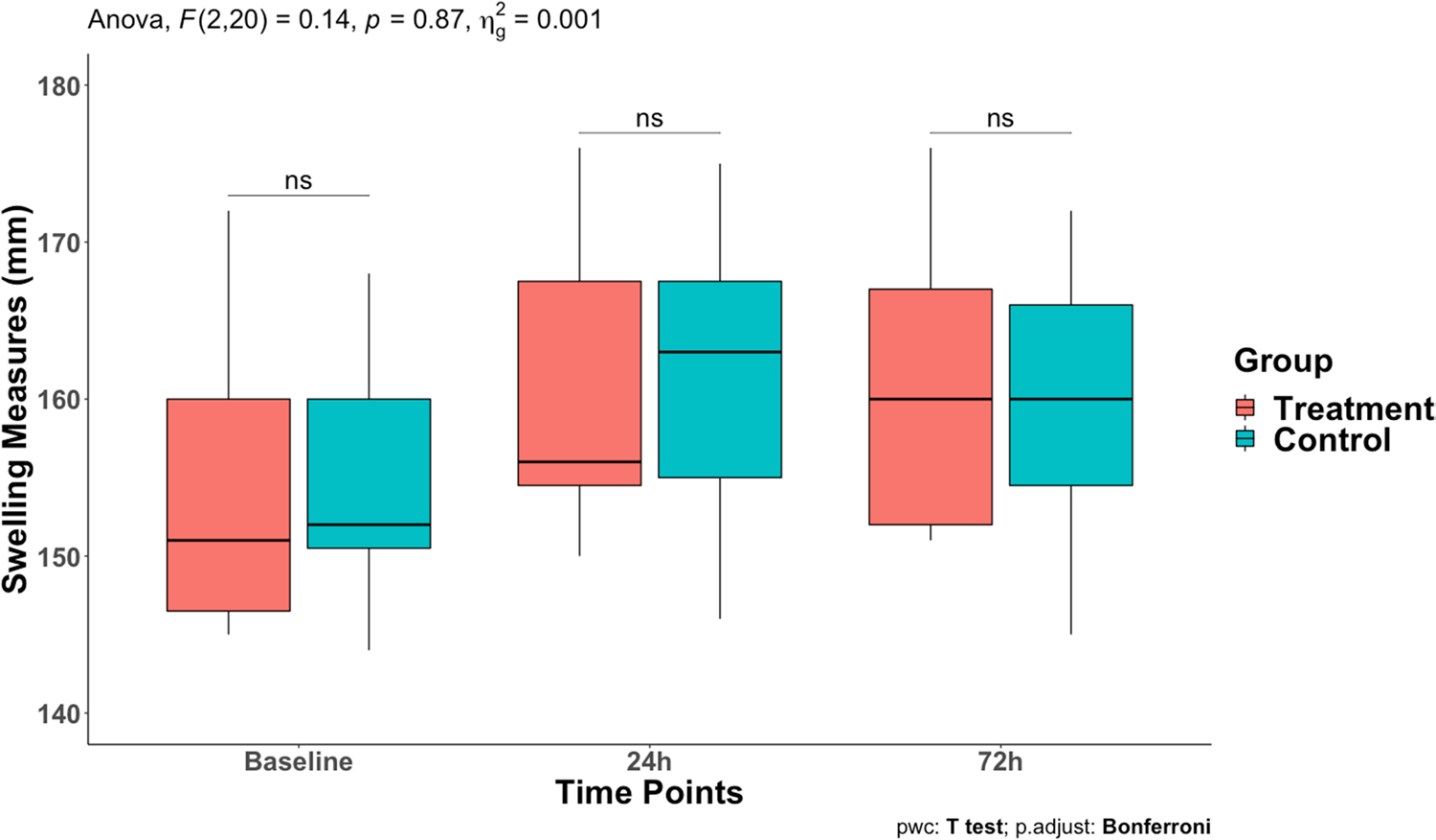
Postoperative swelling repeated measures boxplot between treatment and control groups

**Fig. 3:**
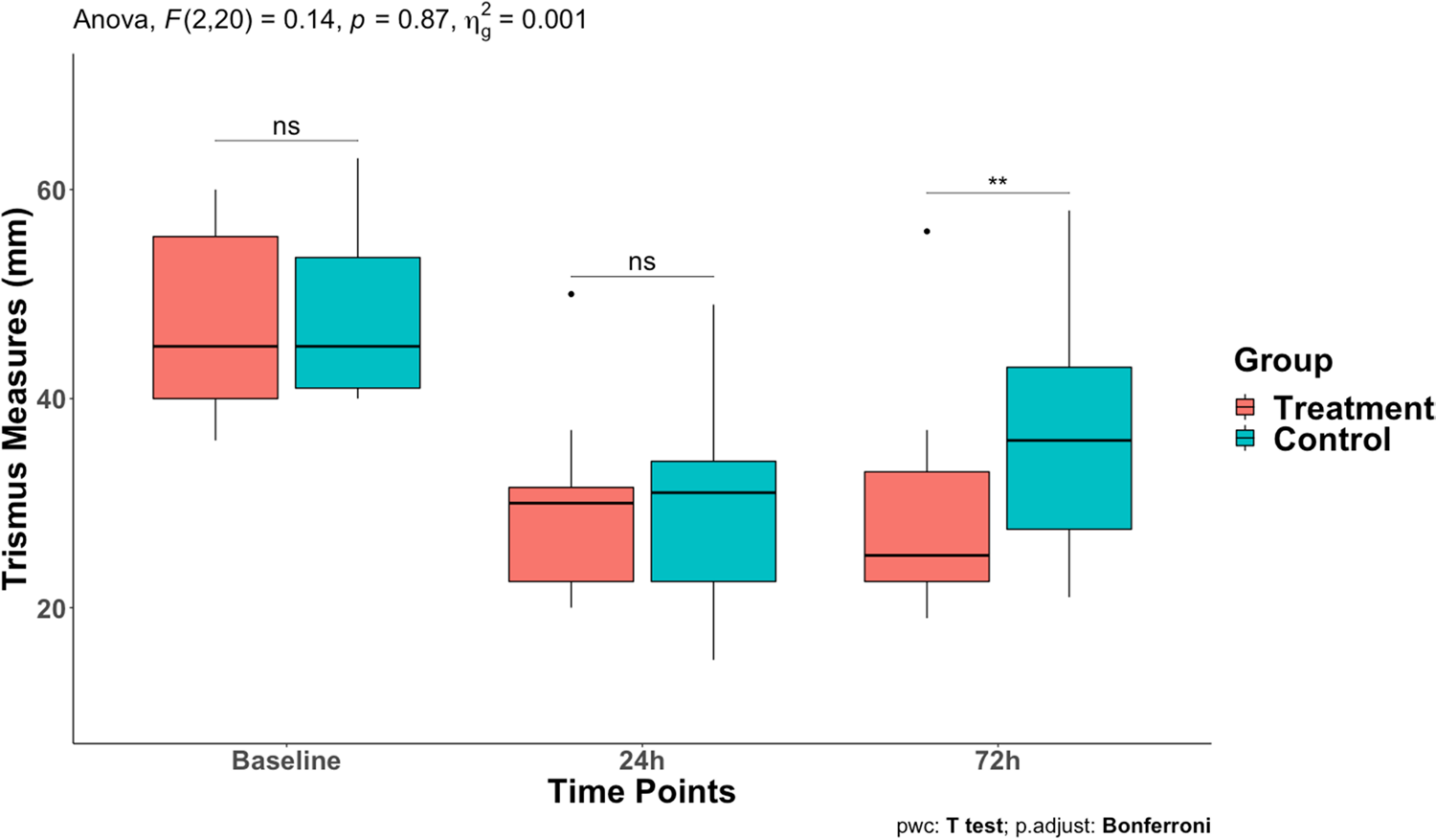
Postoperative trismus repeated measures boxplot between treatment and control groups

Table 2 presents postoperative clinical variables. Regarding our primary outcome, there was no significant difference of VAS pain scores between groups at 24 h, 48 h, and 72 h postoperatively (*p* > 0.05) (Fig. 1). All patients reported that they stopped feeling pain between day 3 and day 5 after surgery with no significant difference between groups (*p* > 0.05). Similarly, we also found no significant difference in total RM consumption (*p* > 0.05). In accordance with our secondary outcome, both facial swelling and mouth opening measures (24 h and 72 h postoperatively) were similar in experimental groups (Figs. 2 and 3, respectively). However, patients in the control group had less restricted mouth opening 72 h postoperatively in comparison to the treatment group (*p* = 0.008). No pharmacological side effects were reported in the study.

**Table 2:** Postoperative clinical parameters

Variables	Treatment	Control	p*
*n*	11	11	
Primary outcome			
VAS pain 24 h (median [IQR])	5.00 [4.00, 6.50]	4.00 [3.50, 6.00]	0.669
VAS pain 48 h (median [IQR])	3.00 [2.00, 5.00]	3.00 [2.00, 5.00]	1
VAS pain 72 h (median [IQR])	3.00 [0.50, 3.00]	1.00 [1.00, 3.00]	0.510
Secondary outcome			
Swelling 24 h (median [IQR])—mm	156.00 [154.50, 167.50]	163.00 [155.00, 167.50	0.929
Swelling 72 h (median [IQR])—mm	160.00 [152.00, 167.00]	160.00 [154.50, 166.00]	0.624
Trismus 24 h (median [IQR])—mm	30.00 [22.50, 31.50]	31.00 [22.50, 34.00]	0.823
Trismus 72 h (median [IQR])—mm	25.00 [22.50, 33.00]	36.00 [27.50, 43.00]	**0.008**
Rescue medication (RM) consumption			
Total RM pills (median [IQR])	1.00 [0.00, 2.50]	0.00 [0.00, 1.00]	0.396
Other pain measures			
No pain—day (median [IQR])	4.00 [3.50, 5.00]	5.00 [3.50, 5.00]	0.529

^*^Wilcoxon signed rank test

## Discussion

The aim of this pilot study was to evaluate the effect of the NSAID chronotherapy in the postoperative period after surgical extraction of third molars, comparing swelling, trismus, and pain.

As it is known, chronobiology is a medical discipline that was first described in the 1960s decade. Its study involves the body rhythms that regulate different functions and are known as circadian rhythms [34, 35]. These circadian rhythms are 24-h cycles that modified the human body condition according to the function and daytime, in two different phases: an active or diurnal and a breaking or nocturnal phase [15].

The influence of circadian rhythms on metabolic processes and their ability to modify the pharmacokinetics of drugs has been described. They can modify the efficacy or side effects of drugs depending on the time of day they are taken; this phenomenon is known as chronotherapy [26]. Chronotherapy has been shown to be efficient in different medical conditions by matching drug administration with circadian rhythms. For example, in the hypertension treatment, the nocturnal intake of the drug has demonstrated to reduce cardiovascular risk [36, 37]; or in oncologic patients, the radiotherapy effect is more efficient, and the side effects are fewer when it is administered during the morning [38]. More recently, it has been hypothesized that the regulation of anti-inflammatory drugs in SARS-CoV-2 patients according to circadian rhythms could result in beneficial management of these patients [39]. Also, one study on asthma patients treated with corticosteroids showed that its administration in the evening provide benefits compared to the morning administration [40].

Lower third molar surgery (LTMS) is, irremediably, associated with pain, swelling, or/and trismus [1]. These postoperative complications can be treated with a wide range of drugs including analgesic and antipyretic (acetaminophen), NSAID (ibuprofen, dexketoprofen), corticosteroids, and opioids [41]. Therefore, many studies have focused on evaluating what would be the best therapeutic option in these cases.

Regarding the trismus associated with postoperative LTMS, Saez et al. compared a chitosan gel against a placebo, observing a lower limitation in the experimental group [42], unlike other authors who used cryotherapy [43], or herbal extracts composed of drugs [44], who did not observe differences between the groups. This clinical trial reported differences significantly lower in the control group than in the experimental group at 72 h postoperatively (*p* = 0.008).

Swelling appears in the soft tissues after an injury or an intentional aggression as the LTMS, according to the immunity response. The tissular damage promotes the prostaglandins releasing and facilitating macrophages or interleukin appearance which works on tissular damage reparation and pain management [45]. There is no evidence about the role of chronotherapy on the swelling or pain after LTM extraction, but the literature is extensive comparing different drugs, dosages, or even combination of different drugs: for example, it is seen that the combination of ibuprofen with acetaminophen report a better post operatory in terms of swelling or pain sensation comparing to a placebo or drugs prescribed isolated [10, 46, 47]. This pilot study, with an alternative ibuprofen administration considering the circadian rhythms, had a positive effect in swelling and pain management, finding no statistical differences about the experimental and the control group in the immediate post operatory and 72 h. Under experimental conditions, the pain sensitivity is higher during the afternoon hours, so that the ibuprofen administration according to the chronotherapy with the morning and afternoon dosage should be enough to maintain a prolonged analgesia, reaching it maximum concentration in plasma when inflammatory mediators reach its peak [39].

Recently a similar study was performed comparing the effect of chronotherapy of the NSAIDs, finding no differences in terms of trismus or swelling indicator and pain scores between the experimental and the control group. They also considered that the night intake of NSAID do not provide any potential benefit in the post-surgical lower third molar treatment [48].

Nevertheless, in the present pilot study, the patients referred a higher pain intensity during the morning or the first hours in the afternoon. This difference was statistically significative (*p* < 0.05) in the experimental group, and it could hypothesize that the patients who did not received the NSAID in the nighttime suffered a higher accumulation of inflammatory mediators that could be traduced in a higher morning pain intensity. Also, though the time between both extraction is 1 month, according to the US Food and Drug Administration, 1 week is time enough for body washing up [49].

One of the strengths of this study is the split-mouth design, thus avoiding interindividual bias in the assessment of the different parameters studied; however, although the results observed in this study are positive, there are some limitations such as the randomization process and the necessity of implement a higher sample size to guarantee the results. Moreover, it should be interesting to compare the chronotherapy by investigating the inflammatory markers levels in blood samples and different types of NSAID with other dosages.

## Conclusion

The use of ibuprofen mediated by chronotherapy has shown similar results to the classical dosage in terms of pain, swelling, or trismus after surgical third molar extraction.

## Data Availability

Data and R Script are available upon request from the corresponding author.
